# Evaluating others’ well-being: Survey experiment on fictional Japanese celebrities generated from wikipedia articles and ChatGPT

**DOI:** 10.1371/journal.pone.0340627

**Published:** 2026-03-12

**Authors:** Takaharu Saito, Aguru Ishibashi, Zeyu Lyu, Zhemeng Xie, Sachiko Yasuda, Hiroki Takikawa

**Affiliations:** 1 Nagoya University of Commerce and Business, Japan; 2 The Institute of Statistical Mathematics, Japan; 3 Tohoku University, Japan; 4 Momoyama Gakuin University, Japan; 5 The University of Tokyo, Japan; Polish Academy of Sciences: Polska Akademia Nauk, POLAND

## Abstract

Well-being attracts scholars’ and policymakers’ interests for decades. This study examines how respondents evaluate a “Good Life,” “Happy Life,” and “Meaningful Life” through the analysis of fictional celebrity articles using the supervised Indian Buffet Process (sIBP). We identify key patterns in well-being perception, highlighting the importance of artistic engagement, public influence, and career success across all three dimensions. While happiness is closely linked to career achievements and personal stability, meaning is driven by cultural and artistic contributions, and a good life balances both elements. Personal hardships negatively impact all three dimensions but are particularly detrimental to happiness. Conversely, creative contributions and public engagement enhance perceptions of a meaningful life. These findings suggest that external success and intrinsic fulfillment are both essential for well-being. Our approach demonstrates the value of computational text analysis in uncovering nuanced insights into societal conceptions of a fulfilling life, paving the way for further interdisciplinary research on well-being and perception.

## Introduction

Well-being has been a central concern for philosophers since antiquity, with both ancient and contemporary thinkers exploring its nature and determinants [1–5]. In recent years, interest in well-being has expanded beyond philosophy, drawing significant attention from policymakers, researchers, employers, and governments [5–10]. This shift has generated a growing body of research seeking to understand how ordinary people evaluate well-being, often through experimental and survey-based methods.

Although well-being is a notoriously difficult concept to define [11], the literature commonly distinguishes among three major dimensions: subjective well-being [3], eudaimonia [12,13], and meaning in life [14]. Building on these conceptual foundations, empirical scholars have explored how ordinary people understand and evaluate well-being, often through survey experiments. For example, college students’ ratings of happiness and meaning in life correlated with their assessments of how desirable and morally good a person’s life was, whereas ratings of wealth (above a certain level) did not [15]. Similarly, moral judgment plays a significant role in attributions of happiness [16,17]. Furthermore, lay conceptions of well-being typically combine happiness, desired satisfaction, and positive objective conditions [18].

Against this background, our study does not attempt to adjudicate between competing philosophical theories of well-being. Instead, we build on the distinctions emphasized in the literature and align our analysis with three corresponding dimensions. A happy life corresponds to subjective well-being, grounded in life satisfaction, pleasure, and the quality of experiences [11,19,20]. A good life reflects eudaimonia, emphasizing vitality, growth, and flourishing [13,21]. Finally, a meaningful life captures the dimension of meaning in life, highlighting purpose, legacy, and existential significance [14].

A major strand of this literature has relied on vignette experiments, in which participants evaluate hypothetical individuals described in short, controlled scenarios. These studies have provided important insights by manipulating pre-defined factors such as subjective versus objective well-being, professional background, or moral character [18,22,23]. Yet, vignette designs also face clear limitations. Because researchers must specify in advance which attributes to include, the range of factors considered is necessarily narrow. Moreover, vignette texts are typically short, stylized, and decontextualized, limiting their narrative realism. As a result, such experiments may underestimate how judgments of well-being emerge in the kinds of rich, media-driven narratives that shape everyday perceptions.

This study addresses these limitations by introducing a novel methodological framework that combines AI-generated biographies with computational text analysis. Specifically, we construct a corpus of approximately 1,200 fictional celebrity biographies written in a Wikipedia-like style. Compared to traditional vignettes, these biographies approximate the narrative form in which the public usually encounters information about others’ lives—through media articles, interviews, and entertainment journalism. While fictional, these texts preserve the length, structure, and detail typical of biographical writing, thereby enhancing ecological validity. At the same time, because the biographies are generated systematically, they allow for large-scale analysis while avoiding the idiosyncrasies of individual real-world figures.

Asking respondents to evaluate fictional celebrities has several benefits. First, this approach allows us to move beyond testing only pre-specified hypotheses and instead examine well-being judgments more inductively. In the existing vignette literature, researchers often construct scenarios by combining predetermined factors such as aspect (subjective or objective), level (high or low), and related attributes (profession, gender, and so on) [18,22,23]. This structure is powerful for hypothesis testing, but it is less suited for discovering which narrative elements people actually attend to when making evaluations. In contrast, our approach is designed to uncover what themes are present in biographies and how they shape judgments across the three dimensions of well-being.

Second, evaluating celebrities’ well-being is a familiar and socially meaningful judgment for many people. Laypeople often assess others’ well-being through TV programs, Social Networking Service (SNS) posts, and entertainment journalism rather than through short fictional vignettes. Especially in Japan, which we focus on in this study, many TV shows have explicitly framed celebrities’ lives in terms of happiness and life satisfaction. For example, a prime-time daily TV series *Jinsei ga kawaru fukaii hanashi* (*Life-Changing Tales and Insights* in English, URL https://www.ntv.co.jp/fukaii/), which had been broadcast for more than a decade, often featured episodes where the camera followed a celebrity for a day, prompting viewers to contemplate whether the life led by the celebrity was truly happy. Therefore, preparing documents similar to those that audiences commonly encounter is desirable for studying how well-being evaluations are formed in practice.

To analyze how well-being evaluations emerge from these narratives, we apply the supervised Indian Buffet Process (sIBP) [24,25]. sIBP is a text-based causal inference method that extends topic modeling: rather than representing documents as mixtures of probabilistic topics on a simplex, it generates binary latent features that can be interpreted as narrative themes. Crucially, these features are discovered in relation to observed outcomes, ensuring that the identified topics are not only coherent summaries of the text but also predictive of how respondents evaluate a Good Life, Happy Life, or Meaningful Life. This makes sIBP particularly suited for uncovering causal patterns in text where conventional unsupervised topic models fall short.

This study also draws on the literature on celebrity culture [26]. The concept of celebrity is closely tied to the historical development of mass media. With the rise of newspapers, radio, and later television, individuals who appeared frequently in the public sphere came to be recognized as figures of collective attention. Unlike traditional forms of fame, which were often confined to political or aristocratic elites, celebrity emerged as a modern and democratized form of visibility, made possible through the expansion of communication technologies and the growing appetite for stories about personal lives rather than public achievements. In this sense, the celebrity is a cultural product born from media logics, where attention itself constitutes social capital [27–30].

Celebrity is also deeply intertwined with consumer society. The expansion of advertising, branding, and global entertainment industries has positioned celebrities not only as performers or public figures but also as commodities to be consumed. They embody lifestyles, values, and aspirations that are marketed to audiences, thereby shaping consumption practices [30–32]. Celebrities thus provide models for identity construction and symbolic resources for everyday life, linking personal desires to wider cultural and economic structures [33,34]. At the same time, celebrity is not only external spectacle but also something that audiences internalize in highly personal ways. Modern audiences develop a sense of familiarity with celebrities despite never meeting them [35].

In recent decades, research on celebrity has expanded into applied fields such as marketing and consumer psychology. A large body of work investigates whether and how celebrity endorsement affects consumer behavior, brand credibility, and purchase intentions (for review, see [36]). Parallel to this, other scholars have explored the more problematic aspects of celebrity influence, including parasocial relationships, addiction to celebrity news, and obsessive fandom [37]. These studies suggest that celebrity culture not only shapes economic outcomes in markets but also profoundly affects individual psychological well-being, sometimes generating unhealthy attachments or unrealistic aspirations.

People rely on celebrities as reference points in making a wide range of judgments, from lifestyle choices to moral evaluations. In this study, we focus specifically on how laypeople assess whether celebrities are living a good, happy, or meaningful life. By analyzing these evaluations, we aim to uncover broader conceptions of well-being that ordinary people hold, as reflected through their interpretations of celebrity lives. At the same time, the project contributes to the literature on celebrity by shifting attention from the structural and psychological functions of celebrity to the evaluative processes of audiences—namely, the factors that shape how people judge the quality of celebrities’ lives. In doing so, we bring together debates on well-being and celebrity to show how public understandings of a “good life” are mediated through cultural frameworks of fame and recognition.

Our findings show that artistic achievement, public recognition, and career success are consistently associated with well-being, though their relative importance varies across dimensions. Happiness is closely tied to professional success and personal stability, while meaning is more strongly linked to cultural and artistic contributions. In contrast, personal struggles and private life challenges carry strongly negative associations, particularly for happiness.

By moving beyond small-scale, pre-structured vignettes to large-scale AI-generated narratives analyzed with sIBP, this study offers a new lens for understanding how people evaluate well-being. It contributes both methodologically—by demonstrating the value of combining generative AI with causal text modeling—and substantively—by showing that the drivers of happiness, meaning, and the good life are shaped by the media-framed narratives through which lives are commonly understood.

## Method and data

### Articles on fictional celebrities generated from Wikipedia and ChatGPT

In order to amass a diverse range of bibliographic data, we leveraged the repository of Wikipedia articles. Our collection focused on articles related to Japanese public figures, specifically those categorized under the headings of Japanese actors and actresses, comedians, and musicians. We intentionally omitted any entries tagged as Japanese pornographic actors or politicians. The resultant compilation encompassed approximately 25,000 articles pertaining to Japanese celebrities.

Subsequently, we applied sentiment analysis to all acquired articles utilizing the “oesti” package [38] with Python. Sentiment analysis is a computational technique that classifies texts according to their evaluative tone, distinguishing between positive and negative language based on word patterns and statistical models. This automated procedure allowed us to approximate how favorably or unfavorably each celebrity was portrayed in public narratives.

From this distribution, we observed that most celebrity biographies clustered around the middle of the sentiment scale, with relatively few texts at the extremes. To ensure sufficient variation in subsequent well-being evaluations, we retained three balanced subsets: 400 articles with the highest sentiment scores, 400 with the lowest sentiment scores, and 400 randomly sampled from the middle range. This approach prevented the corpus from being dominated by mid-range biographies, which would have reduced the variance in perceived well-being outcomes. In total, we obtained a balanced corpus of 1,200 articles. We also note that this sample size was chosen to parallel the literature’s application of the sIBP to 1,286 congressional candidate biographies, thereby enhancing comparability [24].

Furthermore, to circumvent potential criticism associated with the ethical implications of assessing real individuals’ well-being, we employed a novel approach: we transformed the collected Wikipedia articles into fictionalized bibliographies. This was achieved through the use of ChatGPT, which allowed us to generate synthetic profiles while preserving the structural integrity of the original data. We provided the following prompt: “Please create a fictional story inspired by each of the following celebrities’ stories. As a condition, make sure to change all character names, organization names, and other proper nouns so that it is impossible to fully identify the original celebrities.”

Then, we recruited several research assistants and asked them to review each generated article. We asked them to review and revise each of the generated articles in accordance with the following points. Firstly, we addressed errors or highly probable mistakes concerning politically significant issues. For example, one of the generated articles said that Kagoshima was occupied by the U.S., which was incorrect. Secondly, we corrected statements that could potentially affect the reputation or assessment of real celebrities. For example, we revised the texts about marriage and crime. Thirdly, we amended parts that felt unnatural within the flow of a Wikipedia article, such as phrases indicating fictionality or stating “this story is”. Fourthly, we removed descriptions of personal physical features, such as a woman’s body measurements, or excessively sexualized content. Fifthly, we revised deeply rooted episodes concerning real celebrity groups or organizations. Lastly, we corrected any other matters that, to the best of the Research Assistants’ knowledge, contradicted the facts. An example of the experimental stimulus is provided in Supporting Information 1, and the full corpus of generated articles is available at https://doi.org/10.5281/zenodo.18676566.

### Online experiment and quantitative text analysis

Then, we implemented an online experiment. We recruited a sample of 6,000 participants using Cross Marketing, one of the online survey platforms in Japan, on June 12, 2024. Specifically, we recruited 500 respondents for each of twelve demographic groups, defined by sex (male or female) and six age brackets (20–29, 30–39, 40–49, 50–59, 60–69, and 70–79), thereby ensuring a balanced sample across these categories. Before conducting the survey experiment, we submitted a research ethics application to the University of Tokyo’s Experimental Ethics Review Committee and obtained approval for the study (Approval ID: UTSO-23001). Participants were presented with an online explanation of the study’s purpose and procedures, and informed consent was obtained electronically via a checkbox before they began the survey. No personally identifiable information was collected.

At the beginning of the survey, participants were informed that the six celebrities described in the materials were entirely fictional and unrelated to real individuals or organizations. Because this information was provided in advance, no additional debriefing was conducted after the experiment. We considered this procedure sufficient to avoid any risk of participants leaving with false beliefs, and the design was approved by the ethics review. In addition, we carefully revised the stimuli to remove any real celebrity names, ensuring that all characters were entirely fictional. Sensitive details, such as descriptions of women’s physical features, were also deleted following discussion among the co-authors. These steps were taken as part of our ethical considerations to minimize potential discomfort and to avoid reinforcing stereotypes or false associations.

Although there are many definitions of well-being [11,20], in this study we focus on three commonly discussed dimensions of well-being. Respondents were asked to evaluate six fictional celebrities on whether they were living a “happy life,” a “meaningful life,” or a “good life.” These measures correspond to different traditions in the well-being literature: a happy life reflects subjective well-being, focusing on overall evaluations of life satisfaction and affect; a meaningful life captures the presence of meaning and purpose in life; and a good life corresponds to eudaimonia, emphasizing personal growth and flourishing.

The responses were recorded on a five-point Likert scale: (1) strongly disagree, (2) somewhat disagree, (3) neutral, (4) somewhat agree, and (5) strongly agree. Participants evaluated fictional celebrity profiles written in a biographical style; an example profile is provided in the Supplementary Information. All respondents answered the three evaluation questions in the same fixed order (“good life,” “happy life,” then “meaningful life”), and the framing was not randomized across participants or profiles. This resulted in a total of 36,000 observations.

We split the data into a training set (28,800 responses) and a test set (7,200 responses). To analyze the causal relationship between the topics in the fictional celebrity articles and respondents’ evaluations of their well-being, we applied the sIBP [24,25] with R. Unlike traditional topic models such as LDA, which represent topics as proportions constrained to a simplex, sIBP assigns each document a binary vector of latent features. This avoids the interpretability issues that arise when treatments are interdependent, and instead allows us to estimate the independent causal contribution of each discovered feature. Moreover, sIBP incorporates information about the observed outcomes when identifying latent features, ensuring that the topics uncovered are not only coherent representations of the text but also predictive of the responses of interest. We trained the model on the training set and then applied it to the test set to evaluate how well the identified features explained variation in participants’ judgments of a good, happy, and meaningful life.

Since the articles were written in Japanese, we used MeCab, a morphological analysis tool, to tokenize the text. We then transformed the text into a document frequency matrix, selecting tokens that appeared in at least 1,000 but no more than 10,000 documents out of the 36,000 total documents.

For the sIBP model, we set the number of topics (*K*) and tuned hyperparameters *α* and *σ*, as well as the number of iterations. We initially experimented with different values of *α* and *σ* and found that specifying 15 topics provided the best interpretability. We then conducted a parameter search, testing *α* values of 4 and 6, *σ* values of 0.4 and 0.8, and iterations from 1 to 8. Based on exclusivity scores, we found that the optimal parameters for all three dependent variables (good, happy, meaningful) were α=6, σ=0.4, and 4 iterations.

Unlike LDA, where it is common to use quantitative metrics such as semantic coherence or exclusivity to guide the choice of topic number, the sIBP is not typically applied with a formal rule for identifying an “optimal” specification. Prior work has instead emphasized the role of qualitative judgment and interpretability in determining the number of topics [24,39]. In our analysis, we compared solutions with 10, 15, and 20 topics. With 10 topics, domains such as music and film, or comedy and film, were merged into single topics, making interpretation difficult. With 20 topics, the solution became overly fragmented, which further reduced interpretability. The 15-topic specification provided the most balanced and substantively meaningful structure, and we therefore adopted it for the final analysis.

**Fig 1:**
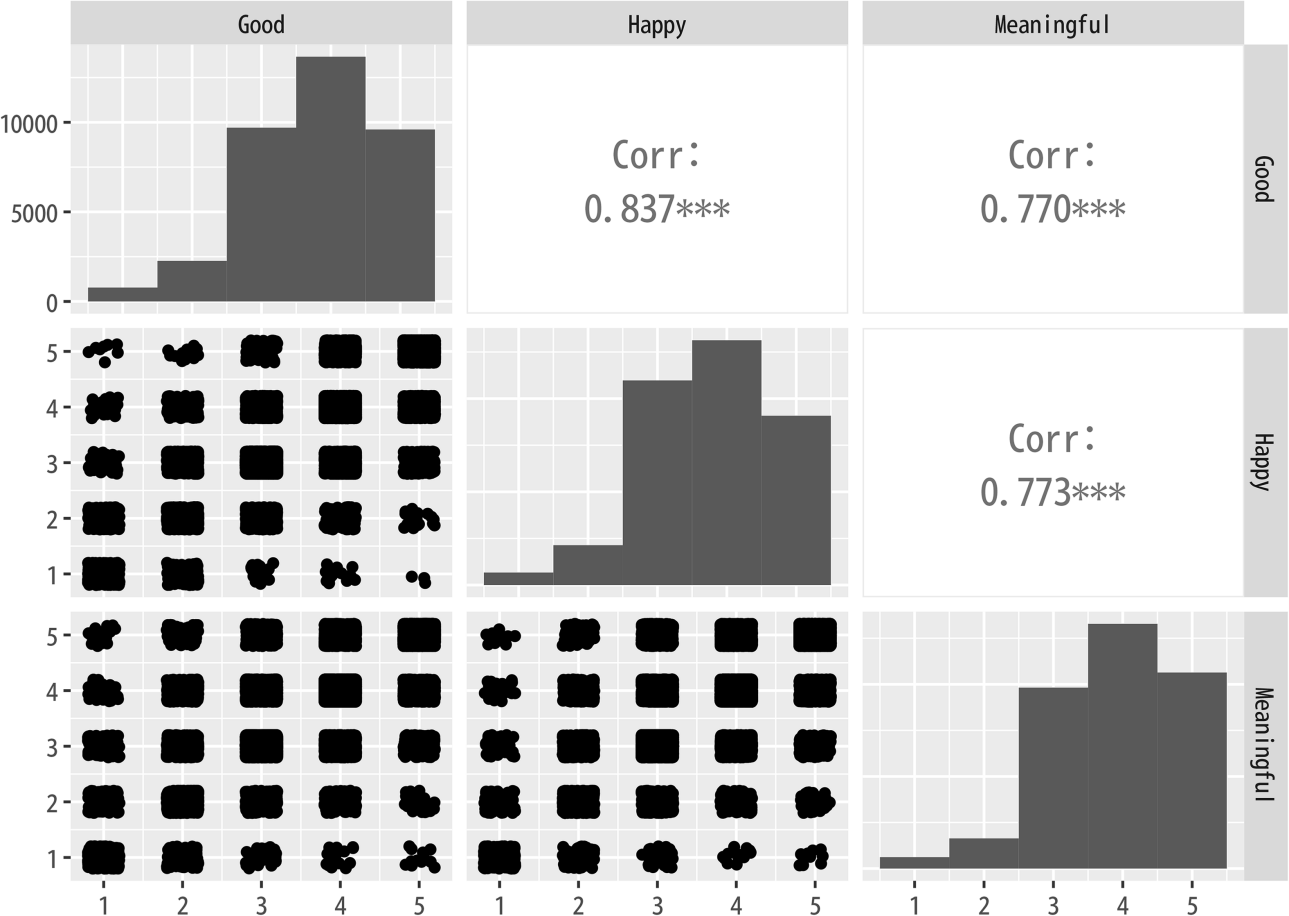
Correlation in Dependent Variables. Good, Happy, and Meaningful refer to respondents’ evaluations of whether the person described in the profile is living a good life, a happy life, and a meaningful life, respectively, measured on 5-point scales.

Using these parameters, we conducted the final sIBP analysis. Tables 1, 2, 3 present the results of the topic modeling component, listing the top 10 words for each topic along with the topic labels we assigned. Notably, the results of the topic modeling component remained largely consistent across good, happy, and meaningful. Since the sIBP topic modeling approach runs separate models for each dependent variable, differences in topic classifications and top words in each topic were expected. However, in this study, participants evaluated the degree to which each fictional celebrity led a good, happy, or meaningful life. As all three measures fundamentally assessed the positivity or negativity of a person’s life, participants’ evaluations were highly similar. Indeed, when calculating the correlations among the three ratings, the lowest correlation was between good and meaningful (0.69), while the highest was between good and happy (0.83) (Fig 1).

**Table 1:** Topic Modeling Results Using sIBP: Analysis of Respondents’ Perception of a “Good Life”.

Topic	Top Words
1. Pre-career	elementary school, public, society, production, environment, resignation, join company, education, issue, photo
2. Media Releases	release, name, place, theme song, disbandment, album, last, single, live, band
3. Early Career	local, song, record, hit, interest, release, high school, talent, junior high, charm
4. Fading Past	join company, ward, death, Shochiku, enjoyment, passing, Kyoto, production, Nikkatsu, Showa
5. Life Change	join company, public, transfer, filming, production, Nikkatsu, place, magazine, same company, marriage
6. Music Industry	single, release, song, hit, music, album, solo, record, singing, audition
7. Comedian Careers	comedian, joke, comedy, duo, video, style, fan, episode, hobby, industry
8. Art	art, power, award, culture, influence, evaluation, training, international, talent, interaction
9. Skills	special skill, successor, resignation, direction, performance, star, voice actor, hobby, joining, like
10. Personal Life	marriage, blood type, wife, type, high school, Nippon TV, divorce, father, store, family
11. Film Production	place, filming, silent, production, Nikkatsu, Meiji, passing, Kinema, joining, Showa
12. Artistic Expression	band, solo, artist, style, impact, successor, later generation, formation, unique, original
13. Public Events	nationwide, event, song, responsible, act, winner, tourism, ambassador, original, movie
14. Comedy Performance	duo, partner, performance, comedy, successor, comedy, dance, comedian, formation, formation
15. Old Film Industry	production, silent, filming, Showa, place, Taisho, death, silent, Meiji, joining

**Table 2:** Topic Modeling Results Using sIBP: Analysis of Respondents’ Perception of a “Happy Life”.

Topic	Top Words
1. Pre-career	elementary school, department, high school, production, part-time job, model, public, famous, work, enrolled
2. Media Releases	release, album, name, theme song, single, live, place, song, star, last
3. Early Career	song, local, record, hit, release, interest, charm, singing, devotion, junior high
4. Fading Past	join company, ward, death, Shochiku, enjoyment, passing, Showa, production, two, Kyoto
5. Life Change	join company, transfer, public, filming, production, Nikkatsu, moment, scout, musical, height
6. Music Industry	single, song, release, hit, music, album, record, style, solo, singing
7. Comedian Careers	comedian, comedy, duo, video, joke, fan, hobby, laughter, channel, interaction
8. Art	art, power, award, culture, influence, evaluation, talent, international, nurturing, performance
9. Skills	special skill, successor, resignation, direction, performance, star, voice actor, hobby, joining, stature
10. Personal Life	marriage, blood type, type, wife, shop, Nippon TV, participation, father, divorce, separation
11. Film Production	filming, place, silent, production, Nikkatsu, Meiji, passing, Kinema, joining, Showa
12. Artistic Expression	band, solo, artist, style, formation, successor, later generation, member, unit, performance
13. Public Events	nationwide, event, song, act, responsible, concert, tourism, ambassador, student, victory
14. Comedy Performance	duo, partner, performance, comedy, successor, comedy, dance, comedian, formation, peer
15. Old Film Industry	production, silent, filming, Showa, place, Taisho, death, silent, Meiji, Kinema

**Table 3:** Topic Modeling Results Using sIBP: Analysis of Respondents’ Perception of a “Meaningful Life”.

Topic	Top Words
1. Pre-career	department, elementary school, production, work, join company, high school, town, birth, passing, public
2. Media Releases	release, album, theme song, name, song, single, song, single, anniversary, star
3. Early Career	local, record, song, hit, interest, charm, release, high school, junior high, talent
4. Fading Past	join company, death, ward, enjoyment, Shochiku, passing, production, Nikkatsu, Showa, one
5. Life Change	join company, public, transfer, production, filming, moment, musical, album, scout, marriage
6. Music Industry	release, single, song, hit, music, album, record, style, solo, singing
7. Comedian Careers	comedian, joke, comedy, duo, video, fan, hobby, laughter, channel, interaction
8. Art	art, power, award, culture, influence, evaluation, talent, international, nurturing, performance
9. Skills	special skill, successor, resignation, direction, performance, star, voice actor, hobby, joining, stature
10. Personal Life	marriage, blood type, type, wife, shop, Nippon TV, participation, father, divorce, separation
11. Film Production	filming, silent, production, Nikkatsu, passing, Kinema, joining, Meiji, location, next
12. Artistic Expression	band, solo, artist, style, formation, successor, later generation, member, development, ambassador
13. Public Events	nationwide, event, song, act, responsible, concert, tourism, ambassador, student, original
14. Comedy Performance	duo, partner, performance, comedy, successor, comedy, dance, comedian, formation, house
15. Old Film Industry	production, silent, filming, Showa, place, Taisho, death, silent, Meiji, same company

Topic labels were generated through an iterative process. The first author initially proposed descriptive labels based on the top words and representative biographies associated with each topic. These preliminary labels were then reviewed and discussed with the co-authors to ensure consistency, clarity, and alignment with the substantive content of the topics. Disagreements were resolved through discussion until consensus was reached. This collaborative procedure helped enhance the reliability and transparency of the labeling process.

## Results

Next, we analyzed the causal effects of these topics on respondents’ evaluations of well-being. As respondents were randomly assigned to articles, potential confounding was minimized. Accordingly, when analyzing the causal effects of these topics on respondents’ evaluations of well-being, explicit adjustment for confounding factors was not strictly required for identification. Nevertheless, we included key covariates—such as gender, age, education level, job type, individual and household income, subjective class perception, parental education levels, and respondent fixed effects—in the regression models to assess the robustness of the results.

### Effect on “Good Life”

**Fig 2:**
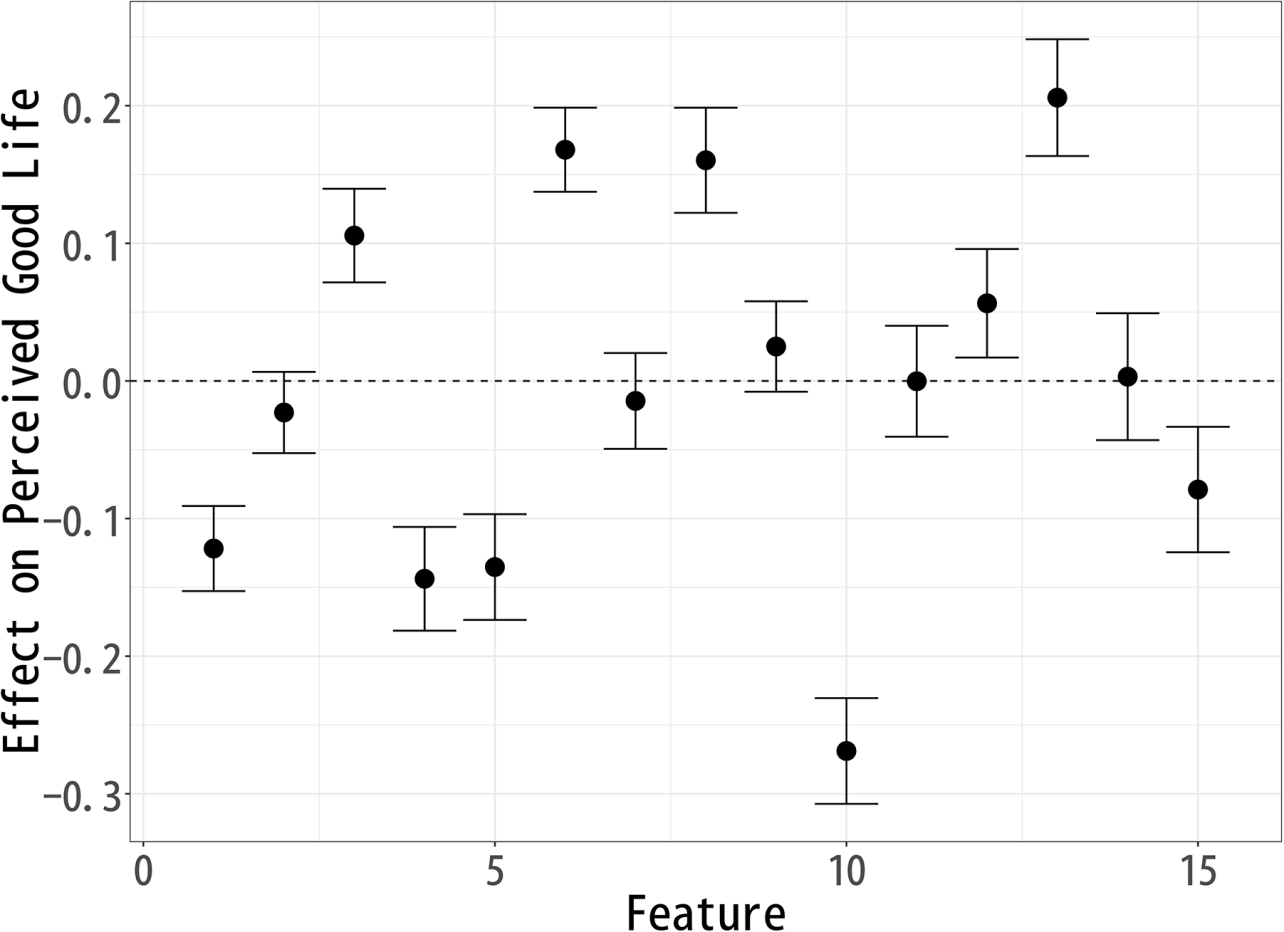
Each Topic’s Effect on Respondents’ Perception of a “Good Life”.

The analysis of topic modeling results using sIBP reveals key patterns in how respondents perceive a “Good Life” after reading fictional celebrity articles. Fig 2 demonstrates the estimated effects of various topics on the perceived evaluation of a good Life. The x-axis represents the different topics (Features) examined in the study, numbered from 1 to 15. The y-axis denotes the estimated effect of each topic on respondents’ perception of a good Life. Positive values indicate that a topic contributes positively to the perception, while negative values suggest a detrimental effect.

The causal effects of different topics on “Good Life” evaluations suggest that respondents associate certain aspects of celebrity life more positively than others in terms of goodness. Topics 3 (Early Career), 6 (Music Industry), 8 (Art), 12 (Artistic Expression), and 13 (Public Events) exhibit a significant positive effect on “Good Life” evaluations. Among these, Topic 13 (Public Events) has the largest positive impact. This suggests that respondents view active public engagement, event participation, and social visibility as key indicators of a good life. Additionally, the prominence of Topic 6 (Music Industry) and Topic 12 (Artistic Expression) implies that artistic creativity, musical success, and public artistic contributions are seen as valuable components of a good life. Topic 3 (Early Career) being positively associated suggests that respondents appreciate narratives of growth, perseverance, and youthful ambition as central to a good life. Topic 8 (Art) reinforces the idea that achievements in cultural and artistic domains are perceived as contributing positively to the goodness of one’s life.

Conversely, Topics 1 (Pre-career), 4 (Fading Past), 5 (Life Change), 10 (Personal Life), and 15 (Old Film Industry) show a significant negative effect on Good Life evaluations, with Topic 10 (Personal Life) having the most substantial negative impact. The strong negative association of Topic 10 suggests that personal struggles, relationships, and private matters may be perceived as diminishing the goodness of one’s life, especially when viewed in the context of a public celebrity image.

Additionally, the negative associations of Topics 4 (Fading Past) and 15 (Old Film Industry) likely stem from their inclusion of words such as death, divorce, and separation, which evoke themes of loss, instability, and personal hardship. This suggests that narratives of decline or personal turmoil contribute to lower perceptions of a good life, as they may symbolize emotional distress or career setbacks rather than long-term fulfillment.

On the other hand, the negative association of Topic 5 (Life Change) indicates that major life transitions—whether career shifts or personal transformations—are not necessarily viewed as contributing to a good life, potentially due to the stress and uncertainty they entail. Furthermore, the negative association of Topic 1 (Pre-career) might indicate that early background factors are perceived as less relevant or even unfavorable in the context of later success.

The findings suggest that respondents prioritize public recognition, artistic creativity, and early career growth over private life stability or long-term professional careers in their evaluation of a good life. The most positive topics emphasize external achievements, visibility, and creative expression, whereas the negative topics highlight personal struggles, career pressures, and industry-related constraints. This indicates that public figures are judged in terms of goodness not only by their success but by how they navigate their careers and how much cultural or social impact they generate.

From a broader perspective, these results highlight the societal emphasis on public engagement, creativity, and career dynamism as core components of perceived good life. Conversely, personal life challenges and professional endurance without public-facing success may lead to a diminished perception of a good life. This insight can inform discussions on media portrayals of celebrities and how audiences form narratives about success based on public and private aspects of a celebrity’s life.

### Effect on “Happy Life”

**Fig 3:**
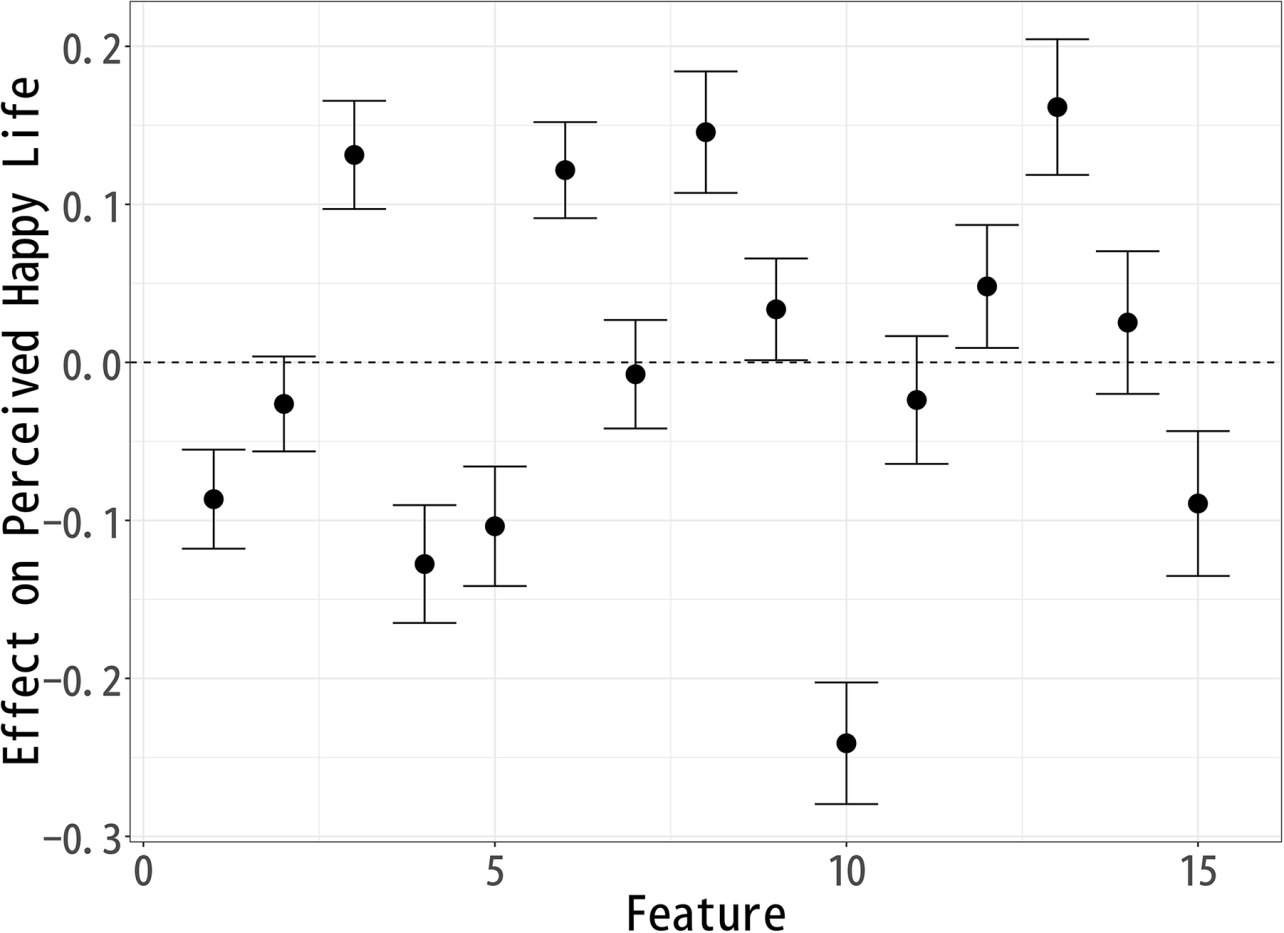
Each Topic’s Effect on Respondents’ Perception of a “Happy Life”.

Building upon the analysis of “Good Life,” we now examine how different topics influence perceptions of a “Happy Life.” Using the same methodological approach, we identify the causal effects of various topics on respondents’ evaluations of happiness. Fig 3 presents the estimated effects, illustrating how certain aspects of a celebrity’s life contribute positively or negatively to perceived happiness. Topics 3 (Early Career), 6 (Music Industry), 8 (Art), 9 (Skills), 12 (Artistic Expression), and 13 (Public Events) exhibit a significant positive effect on “Happy Life” evaluations. Among these, no single topic stands out as having a particularly strong effect, but Topics 3 (Early Career), 6 (Music Industry), 8 (Art), and 13 (Public Events) show similar levels of positive association. This suggests that respondents view artistic engagement, musical expression, and public presence as key contributors to happiness.

The positive impact of Topic 3 (Early Career) indicates that respondents may value personal growth, the excitement of new opportunities, and the energy of youth. Topic 6 (Music Industry) and Topic 12 (Artistic Expression) reinforce the idea that creativity, artistic achievement, and a career filled with artistic milestones contribute positively to happiness. Similarly, Topic 13 (Public Events) suggests that social engagement, participation in public gatherings, and community recognition are perceived as elements of a happy life.

Conversely, Topics 1 (Pre-career), 4 (Fading Past), 5 (Life Change), 10 (Personal Life), and 15 (Film Industry) show a significant negative effect on Happy Life evaluations, with Topic 10 (Personal Life) having the most substantial negative impact. This suggests that personal struggles, relationships, and family matters may be perceived as detracting from happiness, particularly in the context of a celebrity lifestyle.

Additionally, the negative associations of Topics 4 (Fading Past) and 15 (Film Industry) likely stem from their inclusion of words such as death, divorce, and separation, which evoke themes of loss, instability, and personal hardship. This suggests that narratives of decline or personal turmoil contribute to lower perceptions of a happy life, as they may symbolize emotional distress or career setbacks rather than sustained happiness.

On the other hand, the negative association of Topic 5 (Life Change) suggests that major life transitions—whether career shifts or personal transformations—are not necessarily seen as enhancing happiness, possibly due to the stress and uncertainty they entail. Furthermore, Topic 1 (Pre-career) appearing negatively indicates that aspects of one’s early life and background do not play a significant role in perceptions of happiness, or may even be viewed as irrelevant.

These findings suggest that respondents prioritize artistic creativity, personal growth, and public engagement as central elements of a happy life. The most positively associated topics emphasize self-expression, social recognition, and creative pursuits, while the negatively associated topics highlight private struggles, industry pressures, and personal hardships.

This suggests that respondents view happiness in terms of external achievements, visibility, and a dynamic career, rather than private stability or professional longevity. These insights contribute to a broader understanding of how people conceptualize happiness, particularly in the context of public figures, and highlight the importance of cultural and social engagement in shaping perceptions of well-being.

### Effect on “Meaningful Life”

**Fig 4:**
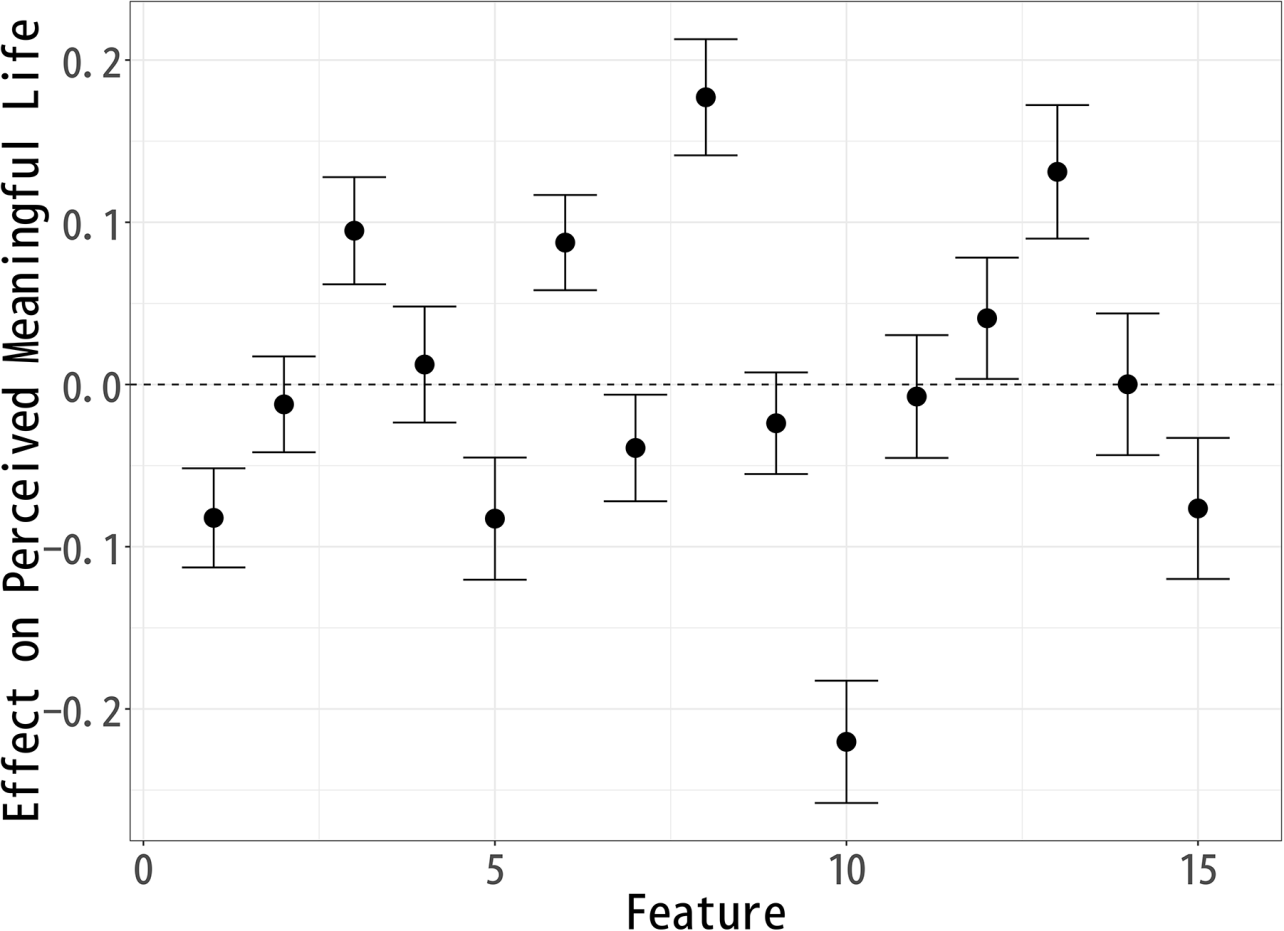
Each Topic’s Effect on Respondents’ Perception of a “Meaningful Life”.

Following the analysis of “Happy Life,” we now investigate how various topics influence perceptions of a “Meaningful Life.” Using the same topic modeling and causal inference approach, we assess which aspects of a celebrity’s life contribute to a sense of meaning. Fig 4 presents the estimated effects, highlighting how different topics either enhance or diminish the perception of a meaningful life. Topics 3 (Early Career), 6 (Music Industry), 8 (Art), 12 (Artistic Expression), and 13 (Public Events) exhibit a significant positive effect on “Meaningful Life” evaluations. Among these, Topic 8 (Art) has the largest positive impact. This suggests that respondents view artistic engagement, cultural contributions, and creative expression as core components of a meaningful life.

The positive impact of Topic 3 (Early Career) indicates that respondents may value the pursuit of aspirations, early achievements, and the process of growth. Topic 6 (Music Industry) and Topic 12 (Artistic Expression) reinforce the idea that music, artistic milestones, and creative endeavors contribute positively to a sense of meaning. Similarly, Topic 13 (Public Events) suggests that community involvement, public influence, and participation in meaningful causes enhance one’s sense of purpose.

Conversely, Topics 1 (Pre-career), 5 (Life Change), 7 (Comedian Careers), 10 (Personal Life), and 15 (Old Film Industry) show a significant negative effect on Meaningful Life evaluations, with Topic 10 (Personal Life) having the most substantial negative impact. This suggests that personal struggles, family-related issues, and private matters may be perceived as detracting from meaning, particularly in the context of a public celebrity image.

Additionally, the negative associations of 15 (Old Film Industry) likely stem from their inclusion of words such as death, divorce, and separation, which evoke themes of loss, instability, and personal hardship. This suggests that narratives of decline or personal turmoil contribute to lower perceptions of a meaningful life, as they may symbolize emotional distress or career setbacks rather than personal fulfillment.

On the other hand, the negative association of Topic 5 (Life Change) suggests that major life transitions—whether career shifts or personal transformations—are not necessarily seen as adding meaning to life, possibly due to the uncertainty and instability they bring. Furthermore, Topic 1 (Pre-career) appearing negatively indicates that past educational background may be seen as irrelevant or less influential in constructing a meaningful life.

These findings suggest that respondents prioritize artistic creativity, public influence, and personal growth as central elements of a meaningful life. The most positively associated topics emphasize self-expression, societal contributions, and engagement in creative and cultural endeavors, while the negatively associated topics highlight private struggles, industry pressures, and professional hardships.

This suggests that respondents perceive meaning in life as being derived from external impact, artistic and cultural achievements, and public engagement, rather than from personal relationships or industry longevity. These insights contribute to a broader understanding of how people conceptualize a meaningful life, particularly in the context of public figures, and highlight the importance of creative expression and societal contributions in shaping perceptions of meaningfulness.

### Robustness checks

In Supporting Information 2, we report a set of robustness checks that assess the sensitivity of our findings to the consolidation of conceptually similar topics. Specifically, we show that merging Topic 7 (Comedian Careers) with Topic 14 (Comedy Performance), and Topic 11 (Film Production) with Topic 15 (Old Film Industry), yields substantively similar sets of top words and preserves the estimated effects on respondents’ perceptions of a “good life,” a “happy life,” and a “meaningful life.” These results indicate that our conclusions are not driven by fine-grained distinctions among plausibly overlapping topics.

### Commonalities and differences

The analysis of topic modeling results using sIBP reveals key patterns in how respondents perceive a Good Life, Happy Life, and Meaningful Life after reading fictional celebrity articles. Since each well-being measure was modeled separately, the topics identified in each analysis are similar but not identical, meaning that direct comparisons across models are not possible. Instead, the results should be interpreted within each individual model, assessing how specific features compare relative to others within Good Life (Fig 2), Happy Life (Fig 3), and Meaningful Life (Fig 4).

Despite these modeling differences, several consistent patterns emerge. Respondents tend to associate artistic achievement, public presence, and personal growth with higher well-being evaluations. Topic 3 (Early Career), Topic 6 (Music Industry), Topic 8 (Art), and Topic 13 (Public Events) consistently exhibit positive effects across all three well-being dimensions. This suggests that creativity, cultural impact, and professional visibility are generally perceived as contributing to overall well-being.

In contrast, personal struggles and private life challenges are universally viewed as detrimental to well-being. Topic 10 (Personal Life) is strongly negative across all three models, with the most pronounced negative effect observed in Happy Life evaluations. This suggests that respondents see personal struggles, family matters, and private relationships as major deterrents to happiness, while their impact on meaning is slightly less severe, possibly reflecting the idea that hardship can still contribute to meaning in life.

At the same time, this finding should be interpreted with caution. In the broader well-being literature, close relationships are consistently identified as one of the most important positive sources of happiness and meaning. By contrast, in our dataset—drawn from media-style biographies—the topic consisted of terms such as marriage, divorce, wife, father, and family, many of which are commonly associated with media coverage of divorce, bereavement, or scandals. As a result, references to personal life carried predominantly negative connotations, reflecting the way intimate life is framed in celebrity narratives rather than its role in everyday well-being. This structural bias helps explain why the topic emerged as a negative topic in our results and indicates that the findings should not be directly generalized to the role of intimate relationships in traditional well-being research. Instead, they speak to media-framed perceptions of well-being, where private matters often become liabilities rather than resources.

Additionally, topics containing words such as death, divorce, and separation—particularly Topic 4 (Fading Past), Topic 10 (Personal Life), and Topic 15 (Old Film Industry)—tend to have significant negative effects across all three well-being measures. This indicates that respondents generally perceive instability and loss as reducing life fulfillment, regardless of whether the evaluation focuses on happiness, meaning, or overall well-being.

Given that the three well-being measures are highly correlated (0.7–0.8), it is important to interpret their differences with care. By introducing the other two measures as control variables in each model, we can examine how topic effects vary once the shared variance across dimensions is accounted for. In this way, the analysis highlights not what is “truly distinctive,” but rather how each dimension of well-being emphasizes certain aspects more strongly than others despite their close interrelation.

**Fig 5:**
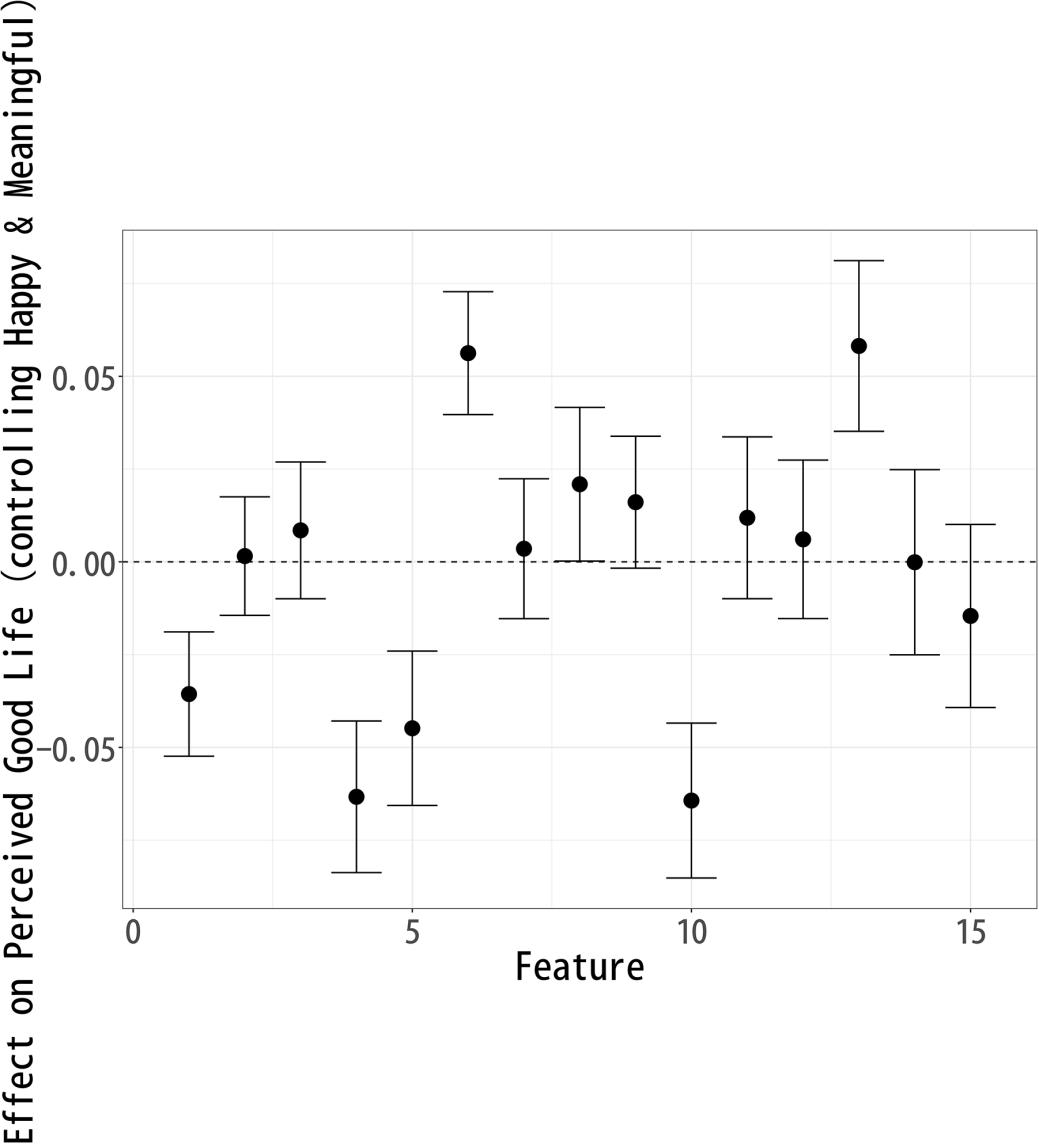
Topic Effects on “Good Life” Evaluations, Controlling for Correlated Well-Being Dimensions.

**Fig 6:**
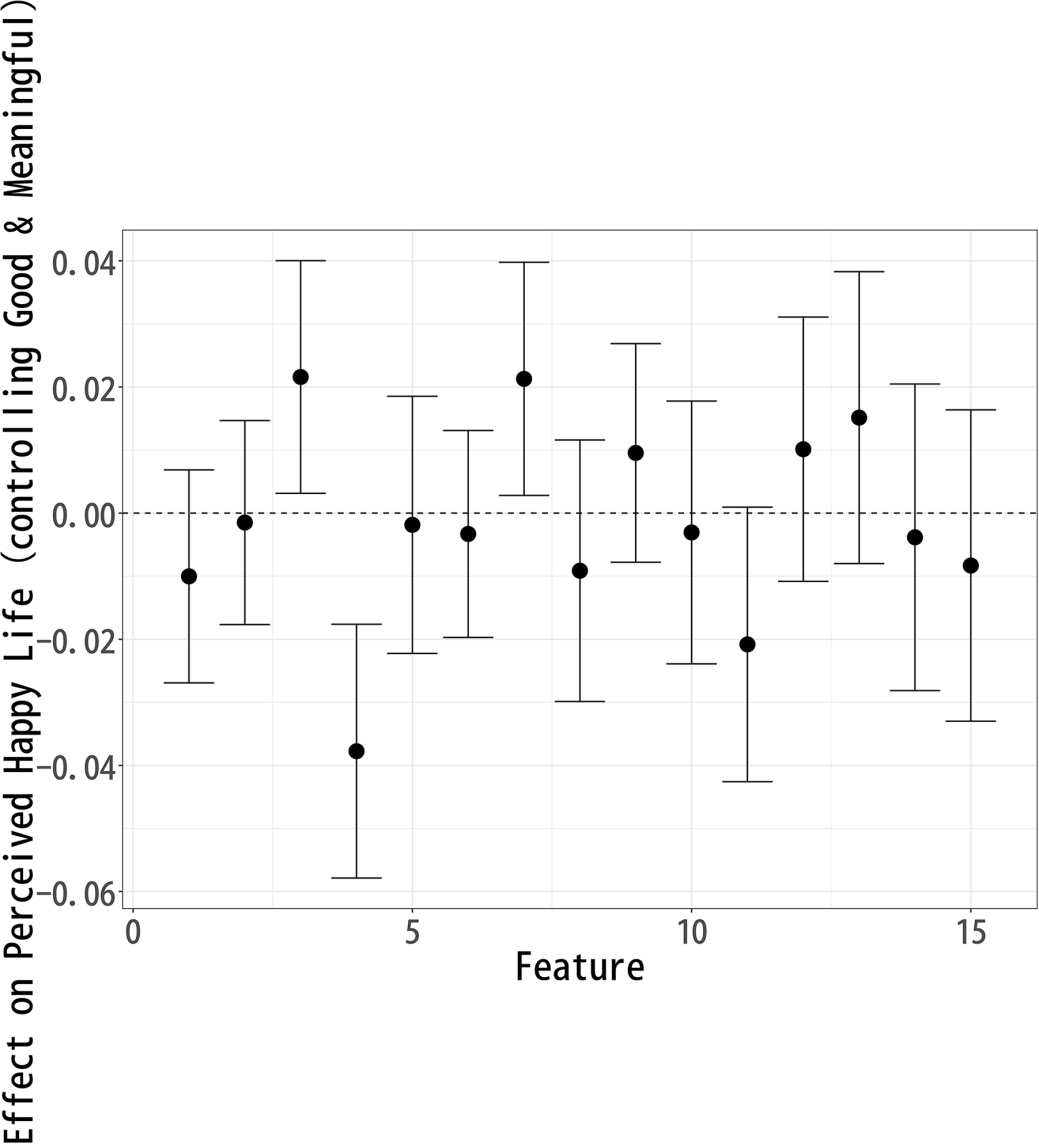
Topic Effects on “Happy Life” Evaluations, Controlling for Correlated Well-Being Dimensions.

**Fig 7:**
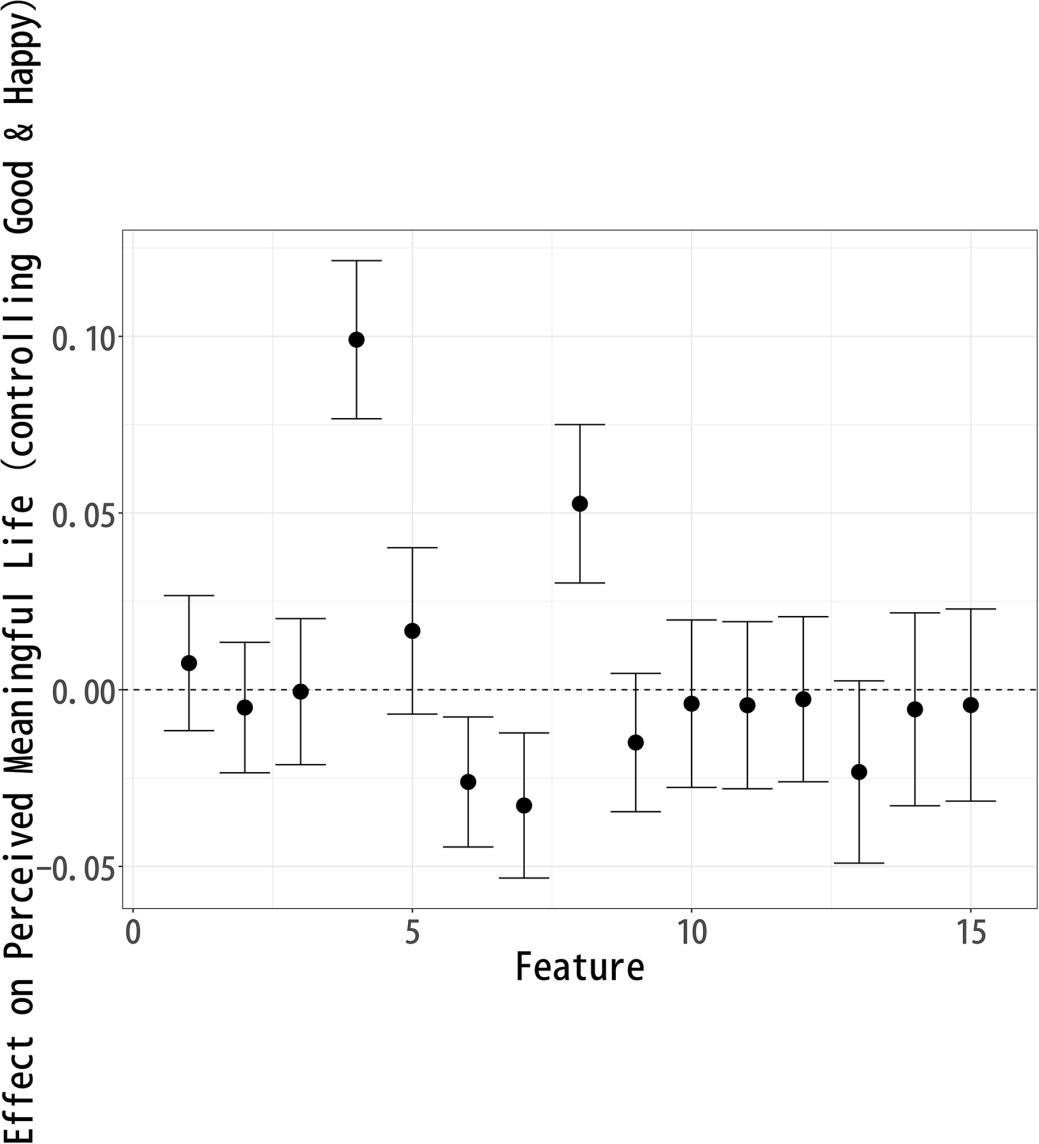
Topic Effects on “Meaningful Life” Evaluations, Controlling for Correlated Well-Being Dimensions.

The topic-level results shown in Figs 5–7 confirm the broader patterns while clarifying these nuanced differences. For perceptions of a good life (Fig 5), topics such as Music Industry (6) and Public Events (13) are positively associated, underscoring the weight of artistic achievement and public recognition. In contrast, topics reflecting private life circumstances or declining careers—Pre-career (1), Fading Past (4), Life Change (5), and Personal Life (10)—show negative associations, indicating that instability and personal struggles diminish evaluations of a good life.

When it comes to happiness (Fig 6), the strongest positive associations appear in Early Career (3) and Comedian Careers (7), suggesting that youthful momentum, popularity, and entertainment value resonate especially with respondents’ judgments of happiness. At the same time, the negative effect of Fading Past (4) highlights that decline or loss of professional vitality is particularly detrimental to happiness.

By contrast, meaningful life (Fig 7) is more closely tied to cultural and artistic contributions. Both Fading Past (4) and Art (8) are positively associated, indicating that even narratives of decline can be endowed with meaning when linked to artistic legacy or cultural memory. Conversely, Music Industry (6) and Comedian Careers (7) are negatively associated, pointing to the perception that commercial success or entertainment careers provide less enduring meaning.

While some topics show consistent associations across all three models, others diverge in ways that reveal the distinctive emphases of each well-being dimension. Topic 8 (Art) plays a particularly strong role in Meaning Life  evaluations, highlighting the view that artistic contributions and cultural legacies are central to perceptions of existential significance. By contrast, Topic 3 (Early Career) is positively associated only with Happy Life, suggesting that youthful momentum and early professional success resonate most strongly with respondents’ sense of joy. Similarly, Topic 6 (Music Industry) is positively linked to Good Life evaluations but negatively associated with Meaning Life, indicating that commercial success is seen as enhancing life achievement and recognition but providing less enduring significance.

At the same time, Topic 4 (Fading Past) illustrates how the same narrative can be evaluated differently across dimensions: references to death and decline are judged negatively when respondents consider a good or happy life, yet these same elements may contribute positively to perceptions of a meaningful life, where legacy and existential reflection play a stronger role.

These contrasts underscore that a good life is evaluated in terms of public recognition and stability, a happy life is tied to vitality and popularity, and a meaningful life is rooted in cultural and artistic contributions—even when intertwined with narratives of decline.

## Discussion

This study contributes to well-being research by employing a novel computational approach to analyze how laypeople evaluate well-being in the context of fictional celebrity narratives. Unlike traditional studies that rely on vignette experiments, which manipulate controlled variables such as profession, morality, and subjective versus objective well-being [18,22,23], our approach examines how individuals naturally assess well-being based on media-driven narratives. Since collective perceptions of well-being are shaped by exposure to biographies, interviews, and entertainment journalism, our method offers a more ecologically valid perspective on how individuals evaluate a good, happy, and meaningful life.

Our analysis reveals that well-being evaluations are shaped by different aspects of celebrity life, with notable distinctions between meaning in life and happiness. While prior research has emphasized the role of morality in meaning in life, our findings suggest that artistic achievement, public recognition, and professional success are more central to perceptions of well-being. Specifically, creative contributions and public engagement were strong predictors of a meaningful life, whereas early career success and involvement in entertainment industries were more closely tied to happiness and general life satisfaction.

A key takeaway is that evaluations of a good life and a happy life tend to move together, both being positively associated with professional success and stability, and negatively influenced by personal struggles. While well-being research often distinguishes eudaimonia from happiness, our findings indicate that happiness and goodness of one’s life—corresponding to subjective well-being and eudaimonia—are largely shaped by similar factors. For instance, career-related achievements and public visibility had strong positive effects on both dimensions, whereas private hardships exerted consistent negative effects, with the most pronounced deterrent impact observed for happiness.

By contrast, meaningful life evaluations show a somewhat different profile. Creative work and artistic expression (e.g., Topic 8: Art) played a particularly strong role in meaning, underscoring the importance of cultural contribution and legacy for existential significance [14]. Narratives of decline or loss (Topic 4: Fading Past) were also evaluated differently across dimensions: for happiness, references to death and decline were perceived as undermining joy and vitality, while for meaningfulness the same elements invoked historical reflection and cultural memory, thereby contributing positively to a sense of purpose and legacy. In addition, commercial success (Topic 6: Music Industry) enhanced perceptions of a good life but was negatively related to meaningfulness, reflecting that entertainment value and professional visibility may provide recognition without necessarily deepening existential meaning.

Taken together, these results suggest that happiness and a good life are grounded in stability, success, and visibility, whereas a meaningful life draws more heavily on artistic legacy and the interpretive value of decline. This highlights that the three dimensions, though strongly correlated, emphasize different pathways through which individuals conceptualize well-being in the context of celebrity lives.

## Conclusion

This study contributes to the growing literature on well-being by applying the sIBP to examine how respondents evaluate different dimensions of life satisfaction in fictional celebrity articles. By analyzing Good Life, Happy Life, and Meaningful Life evaluations, we identify distinct and overlapping factors that shape public perceptions of well-being.

Our findings suggest that artistic engagement, public influence, and career success are key drivers of overall well-being, but their relative importance varies depending on the well-being dimension. While happiness is closely tied to career achievements and personal stability, meaning in life is more strongly influenced by cultural and artistic contributions, and a good life balances elements of both. Additionally, personal hardships negatively affect all three evaluations but are particularly detrimental to happiness, whereas career longevity and industry recognition do not necessarily enhance perceptions of meaning.

However, this study has limitations. Prior research has often examined the relationship between moral virtue and evaluations of a meaningful life, but our analysis could not directly assess this connection. This is largely due to the nature of the dataset, which consisted of Wikipedia-style celebrity biographies and AI-generated extensions. Such texts are a specific genre: they systematically highlight public careers, achievements, and professional milestones, while giving little attention to moral virtues, private relationships, or personal values. As a result, explicit discussions of ethics or character were largely absent from the materials, making it difficult to explore how morality shapes perceptions of meaning. In addition, the focus on Japanese celebrity narratives reflects the cultural norms and media conventions of a specific national context, and thus the findings may not generalize to other societies or cultural settings. Our approach also relies on narrative cues embedded in texts rather than individuals’ self-reports of subjective well-being, which limits the extent to which we can directly capture lived experiences of happiness or meaning. Finally, although we carefully reviewed and edited the AI-generated extensions of the biographies, residual biases inherent in large language models may remain. Taken together, these considerations suggest that our findings reflect how well-being is judged within media-framed narratives of celebrity, rather than offering a comprehensive account of well-being evaluations in everyday social contexts.

These results highlight the need to consider both external achievements and intrinsic fulfillment when evaluating well-being. Future research could further explore how different demographics perceive well-being differently and whether these patterns hold across various cultural contexts. By leveraging computational methods like sIBP, we can continue uncovering the complexities of how individuals conceptualize and evaluate a good, happy, and meaningful life.

At the same time, it is important to emphasize that the present study investigates lay perceptions of well-being rather than its actual determinants. Substantial discrepancies may exist between what people believe makes a life happy or meaningful and the factors that empirically contribute to well-being. Our results therefore should not be interpreted as uncovering the causal sources of well-being, but rather as mapping the beliefs and evaluative frameworks people bring to bear when judging others’ lives through media-style narratives.

## Supporting information

S1 File Example of Fictional Celebrity Profile Used as Ex- perimental Stimulus (English Translation). (PDF)

S2 File Topic Consolidation Robustness Check (sIBP). (PDF)
