# Supplementary material for: Evaluating others’ well-being: Survey experiment on fictional Japanese celebrities generated from wikipedia articles and ChatGPT
Source: PLoS One. 2026 Mar 12;21(3):e0340627. doi: 10.1371/journal.pone.0340627 (PMC12981506; doi:10.1371/journal.pone.0340627)
Supplement: S1 File — (PDF) [file pone.0340627.s001.pdf]

1 Example of Fictional Celebrity Profile Used as Experimental Stimulus (English Translation)

This appendix provides an example of the fictional celebrity profile shown to participants in the survey experiment described in the main text. The profiles were written in a standardized biographical format and were originally presented to respondents in Japanese. The text below is an English translation provided for reference and transparency. The full set of stimuli is made publicly available in https://doi.org/10.5281/zenodo.18676566.

Misaki Haruka is a fictional talent and YouTuber, born on September 12, 1997, in Oyama City, Tochigi Prefecture. She is 168 cm tall and has blood type A. She graduated from the Tokyo Media Arts Academy in March 2016. Her hobbies and special skills include singing, impersonating manga characters, and performing spinning tricks. Her father is a former trainer at the Japan Racing Association (JRA), who retired at the end of 2016. Influenced by him, she developed a deep love for horses. Her given name, Haruka, was inspired by a famous racehorse, Haruka Bijin, which her father had trained. Even her parents' first encounter is said to be related to this horse. She won a special award at the "Sweet Stars Carnival" audition organized by Universal Music, and in August 2014 she joined the group Sweet-hearts as a "new generation Sweet Star," replacing Aimi Sato, who had withdrawn due to health reasons. However, her activities with the group ended later that same year. In 2015, she was selected as one of the top ten finalists in the Oscar Talent Scout Festival, and in 2017, she was also active as a member of Kao Group's "Can-Deli Girls." From August 2021 to April 2022, she performed under the stage name Haruno Yurika, before adopting the name Misaki Haruka on May 1, 2022.
