# Supplementary material for: Evaluating others’ well-being: Survey experiment on fictional Japanese celebrities generated from wikipedia articles and ChatGPT
Source: PLoS One. 2026 Mar 12;21(3):e0340627. doi: 10.1371/journal.pone.0340627 (PMC12981506; doi:10.1371/journal.pone.0340627)
Supplement: S2 File — (PDF) [file pone.0340627.s002.pdf]

2 Topic Consolidation Robustness Check (sIBP)

In topic modeling applications, it is possible that some extracted topics partially overlap in substantive content. In such cases, one may question whether the topic model is effectively serving as a form of dimension reduction, or whether the resulting topic measures may introduce ambiguity in interpretation. Moreover, recent methodological discussions emphasize that topic-based measures used as treatments should be sufficiently distinct to mitigate concerns about confounding due to overlapping or correlated latent dimensions (1).

To address this concern, we conducted a topic consolidation robustness check by merging conceptually similar topics and re-estimating the substantive effects using the same sIBP-based workflow as in the main analysis. We focused on two pairs that appeared particularly close in interpretation: Topic 7 (*Commedian Careers*) and Topic 14 (*Comedy Performance*), as well as Topic 11 (*Film Production*) and Topic 15 (*Old Film Industry*). Using a consolidation procedure that reduces the topic count by one for each merge, we created a consolidated topic set in which each merged topic replaces the corresponding pair. We then re-ran the sIBP-based estimation of topic effects on respondents' perceptions of a "good life," a "happy life," and a "meaningful life," holding all other modeling choices fixed.

Tables 1–3 report the top words of the resulting consolidated topics for each outcome concept. As shown in these tables, the substantive content of the topics remains highly similar to that of the baseline specification. In particular, aside from the mechanically merged pairs, the dominant semantic themes and interpretive labels of the remaining topics are largely unchanged. This indicates that the overall topic structure is stable with respect to reasonable consolidation of closely related topics, and that the baseline topics are not artifacts of overly fine-grained distinctions among semantically overlapping dimensions.

The results are shown in Figures 1–3. Overall, the main substantive conclusions reported in the main article are maintained under this consolidated specification: the direction and broad pattern of estimated topic effects remain consistent with the baseline results, indicating that our findings do not hinge on treating these conceptually similar topic pairs as separate

Table 1: Topic Modeling Results Using sIBP (13 Topics After Merging): Analysis of Respondents' Perception of a "Good Life"

Topic	Top Words
1. Pre-career	elementary school, release, society, production, environment, resignation, join company, education, issue, photo
2. Media Releases	release, credited name, place, theme song, disbandment, album, last, single, live performance, band
3. Early Career	local, song, record, hit, interest, release, high school, talent, junior high, charm
4. Fading Past	join company, ward, death, Shochiku, enjoyment, passing, Kyoto, production, Showa, Nikkatsu
5. Life Change	join company, release, transfer, filming, production, Nikkatsu, magazine, place, same company, marriage
6. Music Industry	single, release, song, hit, musical piece, album, solo, record, singing, audition
7. Comedian Careers	duo, comedy, comedian, partner, joke/material, manzai, formation, laughter, leaving group, performance
8. Art	art, power, award, culture, influence, evaluation, training, international, talent, interaction
9. Skills	special skill, successor (generation), leaving group, direction, performance, star, voice actor, hobby, joining, like
10. Personal Life	marriage, blood type, wife, type, high school, Nippon TV, divorce, father, store, family
11. Film Production	filming, place, silent, production, passing, Showa, Meiji, Kinema, Taisho, join company
12. Artistic Expression	band, solo, artist, style, influence, later generation, formation, performance, development, unique
13. Public Events	nationwide, event, song, responsible, act, championship, tourism, student, ambassador, original

Table 2: Topic Modeling Results Using sIBP (13 Topics After Merging): Analysis of Respondents' Perception of a "Happy Life"

Topic	Top Words
1. Pre-career	elementary school, department, high school, production, part-time job, model, public, famous, work, enrolled
2. Media Releases	release, album, credited name, theme song, single, live performance, place, song, star, last
3. Early Career	song, local, record, hit, release, interest, charm, singing, devotion, junior high
4. Fading Past	join company, ward, death, Shochiku, passing, Showa, production, two, Kyoto, enjoyment
5. Life Change	join company, transfer, public release, filming, production, Nikkatsu, moment, scouting, musical, marriage
6. Music Industry	single, song, release, hit, music, album, record, style, solo, singing
7. Comedian Careers	duo, comedian, comedy, partner, joke/material, manzai, formation, laughter, leaving group, peer
8. Art	art, power, award, culture, influence, evaluation, talent, international, nurturing, performance
9. Skills	special skill, successor (generation), resignation, direction, performance, star, voice actor, hobby, joining, stature
10. Personal Life	marriage, blood type, wife, type, shop, Nippon TV, participation, father, divorce, separation
11. Film Production	filming, place, silent film, production, passing, Showa, Meiji, Kinema, Taisho, join company
12. Artistic Expression	band, solo, artist, style, formation, successor, later generation, member, unit, performance
13. Public Events	nationwide, event, song, act, responsibility, concert, tourism, ambassador, student, victory

Table 3: Topic Modeling Results Using sIBP (13 Topics After Merging): Analysis of Respondents' Perception of a "Meaningful Life"

Topic	Top Words
1. Pre-career	department, elementary school, production, work, join company, high school, town, birth, passing, public
2. Media Releases	release, album, theme song, credited name, song, single, anniversary, star, music, promotion
3. Early Career	local, record, song, hit, interest, charm, release, high school, junior high, talent
4. Fading Past	join company, death, ward, enjoyment, Shochiku, passing, production, Nikkatsu, Showa, early period
5. Life Change	join company, public release, transfer, production, filming, moment, musical, album, scouting, marriage
6. Music Industry	release, single, song, hit, music, album, record, style, solo, singing
7. Comedian Careers	duo, comedian, comedy, partner, joke/material, manzai, formation, laughter, performance, interaction
8. Art	art, power, award, culture, influence, evaluation, talent, international, nurturing, performance
9. Skills	special skill, successor (generation), resignation, direction, performance, star, voice actor, hobby, joining, stature
10. Personal Life	marriage, blood type, wife, type, shop, Nippon TV, participation, father, divorce, separation
11. Film Production	filming, silent film, production, Nikkatsu, passing, Kinema, joining, Meiji, Showa, Taisho
12. Artistic Expression	band, solo, artist, style, formation, successor, later generation, member, performance, development
13. Public Events	nationwide, event, song, act, responsibility, concert, tourism, ambassador, student, originality

dimensions. This suggests that our substantive conclusions are not driven by fine-grained distinctions among highly similar topics, alleviating concerns that topic overlap meaningfully biases the estimated effects.

Figure 1: Each Topic's Effect on Respondents' Perception of a "Good Life" (Consolidated Topics)

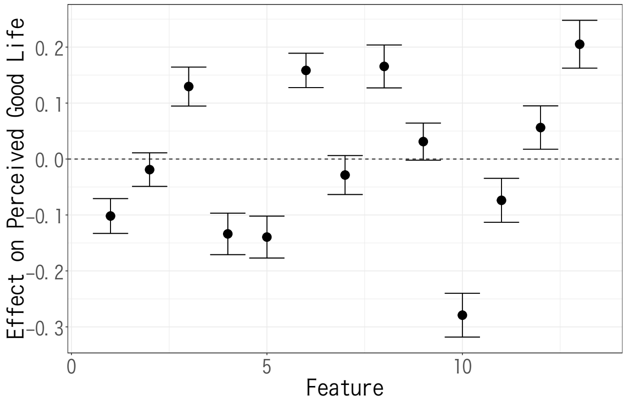

Figure 2: Each Topic's Effect on Respondents' Perception of a "Happy Life" (Consolidated Topics)

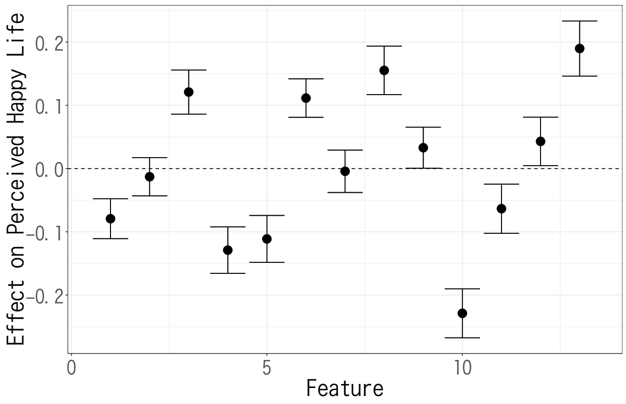

Figure 3: Each Topic's Effect on Respondents' Perception of a "Meaningful Life" (Consolidated Topics)

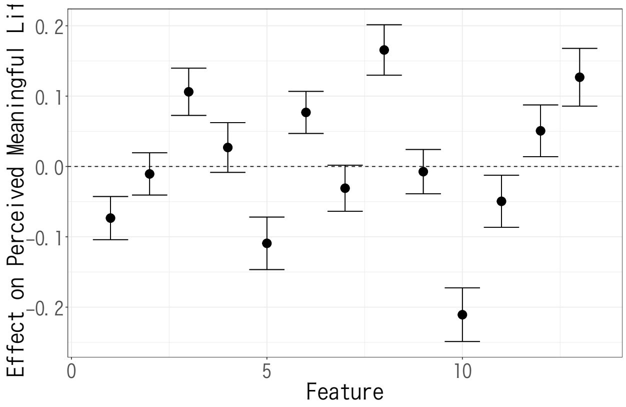

References

[1] Fong C, Grimmer J. Causal Inference with Latent Treatments. American Journal of Political Science. 2023;67(2):374-89. _eprint: https://onlinelibrary.wiley.com/doi/pdf/10.1111/ajps.12649.
